# Strippable Polymeric Nanocomposites Comprising “Green” Chelates, for the Removal of Heavy Metals and Radionuclides

**DOI:** 10.3390/polym13234194

**Published:** 2021-11-30

**Authors:** Gabriela Toader, Daniela Pulpea, Traian Rotariu, Aurel Diacon, Edina Rusen, Andreea Moldovan, Alice Podaru, Raluca Ginghină, Florentina Alexe, Ovidiu Iorga, Sorina Aurora Bajenaru, Mihai Ungureanu, Florin Dîrloman, Bogdan Pulpea, Lucia Leonat

**Affiliations:** 1 Military Technical Academy “Ferdinand I”, 39–49 George Coșbuc Boulevard, 050141 Bucharest, Romania; nitagabriela.t@gmail.com (G.T.); pulpea.daniela@gmail.com (D.P.); andreea.voicu89@gmail.com (A.M.); podaru.alice04@gmail.com (A.P.); mihai.ungureanu@mta.ro (M.U.); florin.dirloman@mta.ro (F.D.); pulpea.b@gmail.com (B.P.); 2 Faculty of Applied Chemistry and Materials Science, University ‘Politehnica’ of Bucharest, 1–7 Gh. Polizu Street, 011061 Bucharest, Romania; aurel.diacon@upb.ro; 3 Research and Innovation Center for CBRN Defense and Ecology, 225 Soseaua Oltenitei, 041327 Bucharest, Romania; ginghinaraluca@gmail.com (R.G.); crinaalexe@yahoo.com (F.A.); iorga_ovidiu@yahoo.com (O.I.); aurorabaj@yahoo.com (S.A.B.); 4 National Institute of Materials Physics, 077125 Măgurele, Romania; lucialeonat@gmail.com

**Keywords:** “green” chelates, complexing agents, nanocomposite, strippable coating, surface decontamination, heavy metal, radioactive material, radionuclide

## Abstract

The issue of heavy metal and radionuclide contamination is still causing a great deal of concern worldwide for environmental protection and industrial sites remediation. Various techniques have been developed for surface decontamination aiming for high decontamination factors (DF) and minimal environmental impact, but strippable polymeric nanocomposite coatings are some of the best candidates in this area. In this study, novel strippable coatings for heavy metal and radionuclides decontamination were developed based on the film-forming ability of polyvinyl alcohol, with the remarkable metal retention capacity of bentonite nanoclay, together with the chelating ability of sodium alginate and with “new-generation” “green” complexing agents: iminodisuccinic acid (IDS) and 2-phosphonobutane-1,2,4-tricarboxylic acid (PBTC). These environmentally friendly water-based decontamination solutions are capable of generating strippable polymeric films with optimized mechanical and thermal properties while exhibiting high decontamination efficiency (DF ≈ 95–98% for heavy metals tested on glass surface and DF ≈ 91–97% for radionuclides ^241^Am, ^90^Sr-Y and ^137^Cs on metal, painted metal, plastic, and glass surfaces).

## 1. Introduction

Over the last decades, many ecosystems have been altered by human activities, causing the contamination of the environment. Heavy metals, as well as radioactive materials, have been extensively used in industrial applications, medicine, military activity, or various research fields. Despite strict loyalty to all laboratory safety procedures, it is still very possible to encounter heavy metal or radionuclide contamination. During the use of materials containing heavy metals or radioactive metals, various surfaces, such as concrete, steel, glass, rubber, plastic materials, or painted surfaces, from a laboratory, a shooting range [1,2], an industrial or a nuclear facility [3,4,5], can be contaminated with these hazardous materials.

“Toxic metals”, including “heavy metals” or “radioactive metals”, are compounds that pose severe environmental problems, negatively affecting the health and the safety of humans at the same time. In very low concentrations, some heavy metals are necessary to support life, but at higher concentrations, they become poisonous due to their bioaccumulation. Occupational exposure to lead is one of the most widespread overexposures. High potential exposures sources include firing ranges, car batteries or pigment industries. Another prevalent heavy metal is mercury. Typical sources of mercury exposure include mining and refining of gold and silver ores. Another category of “toxic metals” is represented by radioactive metals. Radioactive metals are natural or synthetic isotopes of natural non-radioactive metals that can release alpha (α), beta (β), and gamma (γ) radiation [6] In certain circumstances, these metals can be useful for humans, being employed for cancer treatment, material engineering, or for power generation. Uranium is one of the most valuable radioactive material of the modern world. It is the main raw material for nuclear bombs and nuclear power plants. Cesium and strontium are high-yield fission products that are present in significant amounts in fuel pond waters and reprocessing stream liquors [7]. Radioactive contamination can occur as a result of working with radioisotopes, an accident, or even a terrorist attack [8]. A nuclear explosion is followed by the production of a considerable quantity of radioactive cesium isotope ^137^Cs (1.6 times greater than ^90^Sr) [3]. ^137^Cs has long-term consequences due to relatively long half-life [9]. ^90^Sr is utilized in medicine and industry, but it generates significant concerns regarding the fallout from nuclear weapons or nuclear accidents. The probability of ^90^Sr being released as a part of a nuclear reactor accident is lower than the one of ^137^Cs because it is much less volatile, but ^90^Sr is probably the most hazardous element of the radioactive effect from a nuclear weapon.

A major problem with exploiting radioactive metal is represented by wastes. Once released into the environment they will result in possibly catastrophic effects that may last long periods and cause malignant illnesses. While “high-level radioactive waste (HLW)” mainly comes from spent fuel from commercial or research reactors, reprocessing of spent fuel, nuclear weapons, or propulsions industry, “low-level radioactive waste (LLW)” comes from hospitals and industry, as well as the nuclear fuel cycle [6]. External contamination occurs when a heavy metal or a radioactive material, in the form of dust, powder, or liquid, encounters an object or a person. External contamination can become internal if the hazardous material enters their bodies through ingestion, inhalation, or skin. Unfortunately, surface contamination with heavy metals and radioactive metals can, very often, become airborne or can be easily transferred by contact [10].

Considering the significant number of environmental incidents caused by heavy metals or radioactive materials, an overabundance of formulations for decontamination have been developed, and described in the literature, to successfully address different types of contamination scenarios. Efficient decontamination techniques are essential for minimizing occupational exposures, facilitating waste management, restricting the potential accidental release of hazardous materials, and allowing the reuse of some of the components from nuclear reactors, industrial installations, laboratory equipment, shooting ranges, etc. Well-weighted decisions must be taken when choosing between passing the entire system through the decontamination process or replacing just the contaminated equipment/component [11]. Several in situ chemical decontamination technologies, which can be applied for the removal of these hazardous materials, have been developed: wiping with textiles wetted with a decontamination solution [12]; wet vacuum treatment (when the vacuum cleaner is charged with a decontamination solution) [13]; electrochemical decontamination with use of external electrode [14]; foam decontamination [15]; decontamination by etching pastes and gels [16]; decontamination by removable polymer coatings [5,17]; and decontamination by sorbents [16]. A major problem in chemical decontamination is the production of a high volume of secondary waste that needs additional treatment for radionuclides removal. Moreover, to increase the decontamination factor and accelerate the decontamination process, chemical methods involve the use of concentrated acid solutions and temperatures up to 70–90 °C, which could endanger the health and safety of the workers that are manipulating these corrosive and toxic materials. Electrochemical decontamination is limited by the size of the bath in which the contaminated object must be immersed and could not be used on an industrial scale [12]. Using strippable coatings has the advantage of higher efficiency with simpler equipment, fewer chemical reagents, and less waste volume. Among the above-mentioned techniques, decontamination by removable polymer coatings is described in the literature as being the most rapid and cost-efficient technique [16]. Koryakovskiy et al. explained that the application of pastes and gels to the surface takes about 20 to 30 s/m^2^, while their removal takes additional 45 to 90 s/m^2^ or even more to wash the residues; still less productive is an electric brush having a treatment rate of about 8 to 10 min/m^2^ or electrochemical methods, which are expensive and difficult to manage [16].

It is very important to reduce the work time in the contaminated areas because prolonged exposure to these hazardous metals can lead to serious health and safety problems. Moreover, finding a method to remove and fix the contaminants (in a polymeric matrix for example), efficiently with the shortest possible personnel exposure time, is imperative. In this context, removable polymer coatings fulfill most of the requirements listed above. 

This method of decontamination was used for the first time on radioactively hazardous facilities of the naval forces, on nuclear submarines. Since then, polyvinyl alcohol-based decontamination formulations were used on a large scale. These polymeric compositions were employed for Chernobyl accident response activities and displayed the highest decontamination degrees for most of the contaminated materials [16]. 

However, despite the positive findings, certain problems remain regarding the removable polymeric coatings. One of them is related to their chemical composition because some of the film-forming decontamination solutions contain volatile solvents or corrosive components. In addition, they contain “old-generation” chelating agents which are not biodegradable, thus leading to supplementary disposal issues. Another problem is related to their viscosity control, which also needs improvement, because the available commercial solutions have low viscosities and poor adhesion to smooth surfaces and cause serious problems for the decontamination of vertical surfaces, flowing down by gravity. As already mentioned, decontamination of surfaces with strippable coatings is a technique that has been extensively studied in the last decade, due to its multiple advantages, but especially because it is a decontamination method that generates a considerably smaller amount of post-treatment waste [10]. Even though polyvinyl alcohol (PVA), employed in nearly all these types of strippable coatings, is a biodegradable polymer (in specific circumstances), it is still a synthetic polymer and requires special conditions for biodegradation [18]. Moreover, PVA has gained attention lately, being among the major pollutants of industrial wastewater in the textile industry [19]. Therefore, the environmental issues include not only the well-known contaminating agents but also the polymeric materials due to the problems arising from their subsequent disposal. Thus, finding solutions to improve the biodegradability of these decontamination solutions should become a main concern for an environmentally responsible collective attitude. Sodium alginate (SA) could represent an adequate candidate for decontamination applications due to its unique set of properties. SA is a biodegradable hydrophilic linear polysaccharide obtained from marine brown algae [20]. The major advantage of alginates is represented by their liquid-gel behavior in aqueous solutions, due to the ion exchange phenomenon that occurs between the sodium ions in alginate and other divalent ions (calcium ions especially), leading to a gel structure with higher viscosity. In the presence of divalent ions, the G-blocks of alginate participate at the formation of the intermolecular crosslinking, thus gaining enhanced mechanical properties [21]. Depending on the concentration of divalent ions in the system, the crosslinking process can be temporary or permanent. At lower concentrations of Ca ions, the temporary association of the chains can occur, leading to viscous, thixotropic solutions, while at higher Ca ions concentrations precipitation or gelation will occur due to permanent crosslinking phenomena [21]. In the literature, numerous papers have shown that the gel-forming kinetics have a significant influence on its functional properties involving porosity, swelling behavior, stability, biodegradability, gel strength, or biocompatibility [21,22]. Polysaccharides, including cellulose, chitosan, pectin, or alginate, possess the ability to produce films/coatings [23]. Numerous studies have revealed that some films obtained by employing alginate displayed improved barrier and mechanical properties [23,24].

In the actual context, considering all the shortcomings of the existing decontamination methods detailed above, this work describes a novel approach towards obtaining novel water-based film-forming decontamination solutions, containing “green” chelates, for an ecological tactic of efficiently removing heavy metals and radionuclides. These innovative biodegradable solutions can generate resistant and easy-peelable polymeric nanocomposite coatings, due to their specially designed composition, comprising a polymeric blend (polyvinyl alcohol and sodium alginate), with excellent film-forming abilities conjoined with the reinforcing effect brought by bentonite nanoclay. Once these aqueous solutions are applied on the contaminated surface, the complexation of the contaminants occurs, together with their entrapment in the polymer-clay system. After solvent evaporation, they form resistant continuous films that can be easily removed from the surface by simply being peeled off, thus ensuring fast and efficient decontamination. These modern decontamination solutions generate a considerably lower volume of post-decontamination wastes than the traditional methods. The material resulting after decontamination can be easily compacted and temporarily stored in a special small container, until it can be further disposed of as hazardous waste. Another purpose of this study was to evaluate the effect obtained by reducing the PVA amount employed in the strippable coatings through the introduction of SA, an eco-friendlier alternative. Thus, using different amounts of SA, we were able to reduce the PVA concentration in the nanocomposite films while ensuring the same performance: homogenous film-forming ability, ease of film removal, thermal, and mechanical resistance. Another advantage brought by these alginate-based solutions consists of the ability of SA to bind divalent heavy metal ions or radioactive metals, which ensures higher decontamination degrees in this case. Moreover, these novel alginate–based decontamination solutions have higher viscosities that increase in the presence of divalent ions, due to the crosslinking phenomenon, thus allowing the use of these decontamination solutions also on vertical surfaces. Decontamination can possibly occur through two distinct paths: chemical interaction (complexation by “green” chelating agents [25,26,27] or SA), and physical interaction (adsorption by bentonite nanoclay). These two mechanisms, in conjunction with alginate crosslinking [28], enhance the overall efficiency of the decontamination solutions. 

The novelty of this work consists of an innovative way of combining the ability of polyvinyl alcohol to produce films, with the remarkable adsorption capacity of bentonite nanoclay, together with the chelating ability of alginate and “new-generation” “green” complexing agents: iminodisuccinic acid (IDS) and 2-phosphonobutane-1,2,4-tricarboxylic acid (PBTC), to obtain powerful, versatile, and environmentally-friendly water-based solutions for surface decontamination of heavy metal or radioactive metals. Both “green” chelating agents, PBTC and IDS, are well-known in literature for being efficient in chelating metals in aquatic environments [29]. In comparison with the classical complexing agent EDTA, these new generation “green” complexation agents are considered eco-friendly materials, due to their biodegradability. From our knowledge, we report here for the first time, a decontamination solution, containing two “green” chelates: PBTC and IDS, utilized together with sodium alginate as a complexing agent and, at the same time, as an integrant part of eco-friendly peelable polymeric coatings, for the efficient removal heavy metals or radioactive metals from contaminated surfaces. This paper comprises structural, thermal, and mechanical characterization of the newly synthesized nanocomposites through various analytical techniques and decontamination tests on various types of surfaces (glass, metal, painted metal, plastic, and textile sample from the CBRN individual protection equipment) contaminated with heavy metals and three types of radioactive solutions: alpha (^241^Am), beta (^90^(Sr−Y)) and gamma (^137^Cs). 

## 2. Experimental

### 2.1. Materials

Reagent: iminodisuccinic acid (**IDS**—BAYPURE^®^ CX 100 solid G (>78% Iminodisuccinic acid Na_4_ salt, <15% Aspartic acid, Na_2_ salt, <5% Fumaric acid Na2 salt, <0.7% Hydroxysuccinic acid, Na_2_ salt, <0.5% Maleic acid Na_2_ salt and <4% Water)—Lanxess, Cologne, Germany), 2-phosphonobutane-1,2,4-tricarboxylic acid (**PBTC**—BAYHIBIT^®^ AM (40.0–42.5 % PBTC-Na_4_ content in water)—Lanxess, Cologne, Germany), poly(vinyl alcohol) (**PVA** with 98–99% hydrolysis degree, DP ≈ 1700–1800, Mw ≈ 115000 Da—Loba Chemie, Mumbai, India), nano-clay hydrophilic bentonite (**BT**, Sigma–Aldrich, St. Louis, MO, USA), Glycerol ≥99% (**GLY**—Sigma–Aldrich, St. Louis, MO, USA); Sodium alginate (Special Ingredients^®^, **SA**, Garlenda, Savona, Italy). Metal solutions: caesium sulphate (Cs_2_SO_4_—99.99% trace metal basis, Sigma–Aldrich—0.005 M, 0.05 M and 0.5 M Cs_2_SO_4_ aqueous solutions); lead standard for AAS (1000 mg/L ± 4 mg/L Pb in nitric acid, Sigma–Aldrich); strontium standard for AAS (1000 mg/L ± 4 mg/L Sr in nitric acid, Sigma–Aldrich); cobalt standard for AAS (1000 mg/L ± 4 mg/L Co in nitric acid, Sigma–Aldrich). Radioactive solutions: ^137^Cs; ^241^Am; ^90^(Sr-Y). Tested surfaces: stainless steel sheets (10 mm × 10 mm × 0.4 mm classical 18/8 stainless steel), galvanized metal sheets (10 mm × 10 mm × 0.4 mm), brass sheets (10 mm × 10 mm × 0.5 mm) and cooper sheets (10 mm × 10 mm × 0.2 mm), metal (100 mm × 100 mm × 0.5 mm, MIL-46100 military grade steel), painted metal (100 mm × 100 mm × 0.5 mm, paint based on urethane modified resin), plastic (100 mm × 100 mm × 1 mm, polycarbonate), glass (100 mm × 100 mm × 5 mm) and BC/SP2 (Romanian Army Individual Protective Equipment (IPE) material sample (100 mm × 100 mm × 0.5 mm)).

### 2.2. Methods

#### 2.2.1. Synthesis of the Decontamination Solutions

The water-based decontamination solutions were obtained according to the procedure described below. The correlation between the composition of the decontamination solutions and the sample IDs is summarized in Table 1. Every decontamination solution contains water, 5% PVA, 1% BT nano-clay, 2.5% glycerol, and different sodium alginate concentration (0%, 0.25%, 0.5%, 0.75%, or 1%), indicated in Table 1. Each of the last two samples (GD-3-PBTC and GD-3-IDS), contained 1% chelating agent (CA). The aqueous decontamination solutions were obtained through the following steps: the first one consisted in the dissolution of the chelating agent in water (for GD-3-PBTC and GD-3-IDS samples), followed by the dispersion of bentonite by ultrasonication. The next step consisted in the addition of the alginate water solution, followed by the dissolution of PVA. The last step consisted in the addition of glycerol. The solutions were stored at 2–5 °C until they were employed for decontamination tests. All the decontamination solutions containing SA exhibited a liquid-gel behavior in the presence of divalent ions, changing from a relatively low viscosity solution to a gel structure. They were allowed to dry on the target surface, forming thin films, which were subsequently removed by being peeled off. The nanocomposite films were subjected to different analytical investigations, described in Characterization Section 2.3. 

#### 2.2.2. Nanocomposite Films Preparation

To evaluate the properties of the nanocomposite films obtained from the decontamination solutions, various analytic techniques were employed (described in the Characterization section). The films subjected to analysis were obtained through casting method. Decontamination solutions were placed in rectangular glass molds (12 cm × 12 cm × 2 cm), and they were allowed to dry on a plane horizontal surface, at room temperature and 50–55 relative humidity. Usually, the drying time for these film-forming materials is below 24 h, depending on the thickness of the film and the environmental conditions.

#### 2.2.3. Controlled Contamination for SEM-EDS Analysis

Non-radioactive cesium was employed for evaluating in-depth and surface contamination on three different types of metallic surfaces. For this purpose, Cs_2_SO_4_ solutions were employed for the contamination of stainless-steel surfaces (square-shaped metallic coupons: 1 cm × 1 cm × 0.05 cm) with 3 types of finishing: mirror-finish (SSMF), grinded-finish (SSGF), and etched-finish (SSEF)—immersed in royal water). To reproduce in-depth contamination [30], stainless steel coupons were immersed for 30 min of in 0.005 M Cs_2_SO_4_ aqueous solution followed by a thermal treatment at 700 °C, in a furnace, for 2 h. To simulate superficial contamination, three different concentrations of Cs_2_SO_4_ aqueous solutions were employed: 0.005 M, 0.05 M, and 0.5 M. The metallic coupons were placed in Petri dishes and the contamination solution was added until they were completely covered by liquid. Then, the coupons were maintained at 40 °C until the complete evaporation of water. For the decontamination tests, metallic coupon was covered with decontamination solution. Once the drying process (complete evaporation of water, at 25 °C, approximately 8 h) ended, the strippable coatings were easily peeled off from the metallic coupons. Both, polymeric films containing the contaminant and decontaminated surfaces were subsequently subjected to SEM/EDS (Billerica, MA, USA) analysis. This investigation offered preliminary qualitative information about the decontamination process. To quantitatively evaluate the decontamination efficiency, decontamination tests described below were performed.

#### 2.2.4. Decontamination Tests

Decontamination tests aimed to evaluate the removal efficacy of herein reported decontamination solutions. For this purpose, controlled contamination with heavy metals and radionuclides was performed prior to the decontamination step. For the evaluation of the decontamination degree, two distinct analytic investigations were performed: (1) atomic absorption spectrometry and (2) alpha, beta, and gamma radiation measurements. 

#### 2.2.5. Evaluation of the Decontamination Efficacy through Atomic Absorption Spectrometry (AAS) Technique

For the controlled contamination with heavy metal, a standard solution of lead was employed. Standard solutions containing Sr and Co were also utilized for controlled contamination (as simulants for their analogous radionuclides). To obtain surfaces with similar contamination degrees, 1 mL from each metal standard solution (Pb, Sr, and Co) was placed in a distinct glass Petri dish (φ = 50 mm) and they were allowed to dry. Then, the decontamination solutions (8 mL) were applied on the contaminated surfaces. After being completely dried, they were easily peeled. Triplicate experiments were performed for a better accuracy. Decontamination efficacy was evaluated by comparing the remnant metal concentration from the surface with the initial concentration of contaminant. For this analysis, after removing the strippable coatings, the Petri dishes were washed three times with 5 mL of distilled water and the collected solutions (3 × 5 mL) constituted the sample that were subjected to AAS investigation, to assess the remnant metal concentration on the decontaminated surface. The decontamination factor was calculated with the following formula: DF = 100∙(C_0_ − C_f_)/C_0_ [4,30], where DF is the decontamination factor, C_0_ is the initial metal concentration, and C_f_ is the final concentration, reflecting the residual contamination. 

In parallel, the concentration of “toxic metal” found in the polymeric nanocomposite film was also calculated using the AAS technique. For this purpose, each of the polymeric nanocomposite films obtained was dissolved in 50 mL of distilled water, sonicated, and then centrifugated and filtered to obtain a clear aqueous solution that was also subjected to AAS analysis. Since these samples required intermediary operations before being ready for AAS analysis, we can use the concentrations obtained only to demonstrate the presence of the “toxic metal” in the exfoliated film, and we can presume that the lacking amount of contaminant remained fixed in the bentonite eliminated after centrifugation and filtration steps. The differences between the values obtained for “toxic concentration” occur due to a certain degree of uncertainty evaluated internally, for the performance of sample preparation procedures for AAS analysis. This consists in a cumulative uncertainty regarding the measuring instruments, the human factor, and the analysis equipment. Losses also occur in the process of extraction, filtration, and dilution. To minimize the uncertainty, triplicate analyses were performed with the same sample, reporting the measured average value. The data collected from these decontamination tests are useful for a better understanding of the metallic contaminant removal process. As already mentioned above, these experiments were repeated three times and the average value was reported.

#### 2.2.6. Evaluation of the Decontamination Efficacy through Radiation Measurement

Three standard radioactive solutions, ^241^Am for alpha radiation, ^90^ (Sr-Y) for beta radiation, and ^137^Cs for gamma radiation, were employed for radioactive controlled contamination of 5 types of surfaces: metal, painted metal, plastic, glass, and BC/SP2. The radioactive controlled contamination was performed according to NATO standard AEP-58 [4]. Every tested surface measured a 10 cm^2^ area and a specific quantity of each solution was uniformly dispersed on the surfaces to reach a value between 30 ÷ 80 Bq/cm^2^ for medium contamination level and 300 ÷ 800 Bq/cm^2^ for high contamination level. The activity of the contaminated surface was measured at 10 min after the deposition of the radioactive solution. After measuring the initial activity (A_i_) of the surfaces, 10 mL of decontamination solution was poured over the targeted area. The decontaminating solution was allowed to cure and dry. After the completion of this step (after 24 h), the coatings were peeled off and the final activity (A_f_) of the surface was measured again immediately after the removal of the nanocomposite film. The decontamination factor (DF) was calculated considering the initial activity of the contaminated surfaces and the final activity of the decontaminated surface, with the aid of the following formula (equivalent to the one utilized for heavy metals): DF = 100 (A_i_ − A_f_)/A_i_, where A_i_ represents the initial contamination (Bq/cm^2^) and A_f_ represents the residual contamination (Bq/cm^2^), measured after the removal of the nanocomposite film. DF was determined in accordance with NATO standard AEP-58 [4]. The radioactive activity investigations were carried out with Berthold dose/dose rate monitor. The measurements for each type of sample were repeated three times and the average value was reported.

### 2.3. Characterization

FT-IR spectra were obtained with the aid of a Perkin Elmer Spectrum Two with a Pike Miracle™ ATR modulus, at 4 cm^−1^ resolution, 550 to 4000 cm^−1^. Thermogravimetric analysis (TGA) was performed on a Netzsch TG 209 F3 Tarsus instrument (Selb, Germany) under nitrogen atmosphere, a flow rate of 20 mL/min, on samples weighting approximately 4 mg, on a temperature ramp starting from ambient temperature up to 700 °C with 10 °C/min heating rate. A 710 Titan 2 universal strength testing machine, equipped with a 3000 N force cell was employed for tensile tests, performed according to ISO 37: 2011(E). The dumbbell-shaped specimens were obtained from the strippable nanocomposite films using a cutting mold device that had 75 mm overall length. The test area implied a length of the narrow section of about 25 ± 1 mm. The variation of the length and force was continuously monitored with an accuracy of ±0.2% at a speed of 8.33 mm/s. Five specimens from each sample were analyzed. The mean values obtained for each material were plotted in a comparative stress–strain graph. To evaluate thermo-mechanical and viscoelastic properties of the nanocomposite coatings, measurements were conducted on a Discovery 850 DMA-TA Instruments, in single cantilever-bending mode. Experiments were run on samples (10 × 30 × 0.5 mm^3^ size), at a frequency of 1 Hz, and a temperature ramp starting from −80 °C to 200 °C with a heating rate of 5 °C/min. SEM-EDS analysis was performed with the aid of a Zeiss Gemini 500 microscope coupled with an XFlash 6 EDS detector from Bruker (Billerica, MA, USA). All data from the EDS were analyzed using the ESPRIT Software (version ESPRIT 2, Billerica, MA, USA). For the evaluation of the efficiency of heavy metal removal we employed an atomic absorption spectrometer, PerkinElmer AAnalyst™ 800 (Waltham, MA, USA) high-performance with WinLab32 software (Perkin Elmer, Waltham, MA, USA) for AA. The determinations were performed by subjecting samples to electrothermal atomization in a graphite furnace. The radionuclides decontamination efficiency was determined by measuring the activity of the targeted surfaces (contaminated with ^241^Am, ^90^ (Sr-Y), and ^137^Cs), before and after decontamination, using Berthold L123 dose/dose rate monitor with specific detectors for alpha, beta, and gamma radiation. 

## 3. Results and Discussion

**Scheme 1:**
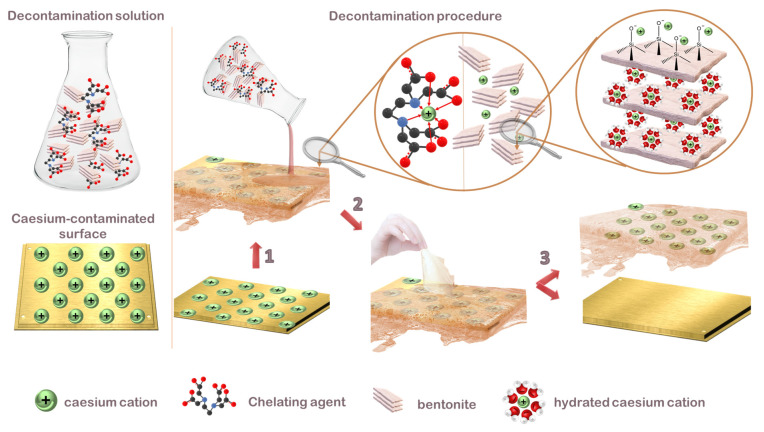
Schematic illustration of decontamination process: (**1**) application of the decontamination solution on the contaminated surface; (**2**) decontamination process (exemplified by complexation or adsorption mechanisms); (**3**) removal of the contaminants entrapped into the nanocomposite film.

The decontamination process with strippable coatings involves the following three steps, presented in Scheme 1: (1) The decontamination solution is applied (poured or sprayed) on the surface contaminated with the “toxic metal” (heavy metal or radioactive metal). For exemplification, Scheme 1 describes contamination with cesium, because ^137^Cs is one of the radionuclides investigated in this study, but it could be replaced with any other metallic contaminant. The decontamination reaction occurs at the interface between the aqueous decontamination solution and the contaminated metallic surface. If this solution maintains its liquid state, contaminant ions are continuously extracted from the moistened metallic surface into the decontamination solution through complexation and adsorption mechanisms. As the solvent (water) is lost or the polymers undergo crosslinking, the metallic contaminants are entrapped inside the matrix of the polymeric nanocomposite. (2) After stage (1) is complete (less than 24 h), the dry nanocomposite film containing the contaminant can be easily peeled off from the metallic surface. The strong interactions between the components of these materials ensure the formation of a compact polymeric nanocomposite film which maintains its integrity when it is detached from the surface. (3) When the peeling process ends, the metallic surface is considered successfully decontaminated. The strippable coating containing the “toxic metals” should be compacted and sealed in a small container towards final disposal. According to legislation requirements, these materials should be disposed or incinerated as low-level waste [31], but their greatest advantage is represented by the small volume of material generated post-decontamination. 

The schematical illustration (Scheme 1) presents only the main mechanisms of action of the active components from our decontamination solution: complexation (by chelating agents) and physical interactions (specific interactions between nano-clay and contaminant cations). Bentonite clay possesses five types of adsorption sites [32]: basal surface site (planar site), interlayer site, hydrated interlayer site, edge site, and the frayed edge site (FES) and their existence allows metal contaminants adsorption through distinct routes [32,33,34], part of them drawn in Scheme 1. The chelating effect of SA is also a factor which influences decontamination performances, but since it did not bring any outstanding improvements, it was intentionally omitted from this scheme, for the ease of visualization.

**Figure 1:**
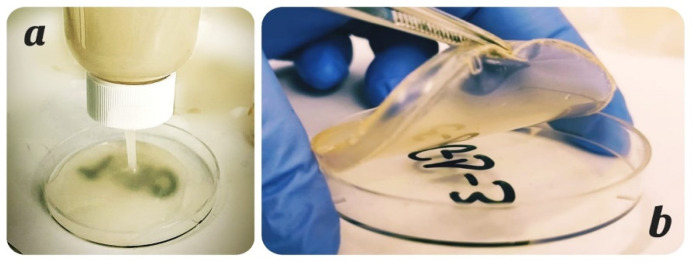
Casting process: (**a**) decontamination solution is transferred on the contaminated surface; (**b**) the surface is decontaminated after film removal.

The nanocomposite strippable coatings were obtained through casting method, as described in Methods Section 2.2. Figure 1 illustrates the casting process. As can be seen in Figure 1a, the decontamination solution can be easily transferred on the contaminated surface. Depending on the surface type and position, these solutions can be simply poured onto the surface, applied with a roller or a brush, or they can be applied by spraying technique. After being deposited on the targeted area, the decontamination process begins. The decontamination solution is allowed to dry and after the complete evaporation of the solvent (water), the nanocomposite film can be peeled off (Figure 1b), and thus the surface is decontaminated. These decontamination formulations are customizable, because the active ingredients of these decontamination solutions can be selected depending on the targeted contaminating agent (chemical, biological, radiological, or nuclear contaminants). In this study, “green” chelating agents and sodium alginate were employed, and the aim was “toxic metals” (heavy metals and radioactive metals) removal. 

The composition of the decontamination solutions and the interactions that occur between the components have a significant influence on the performances of the strippable coatings obtained. Thus, various decontamination formulations comprising different SA concentrations (Table 1) were developed to establish which composition of the polymeric matrix is adequate for this type of application. 

**Figure 2:**
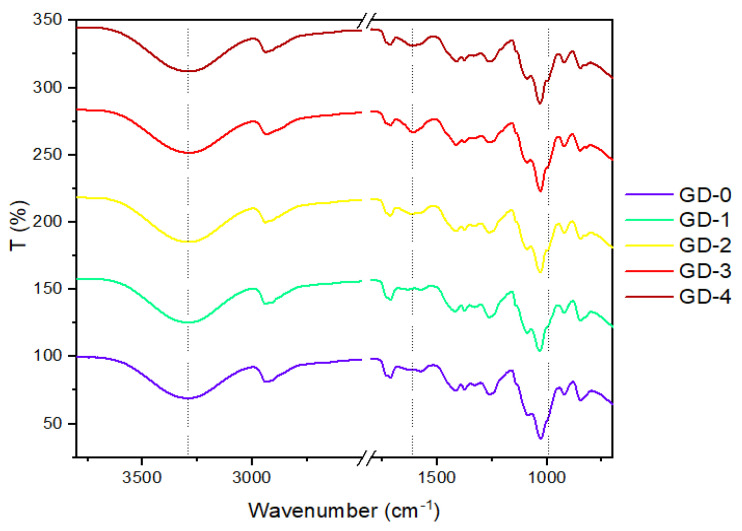
FT-IR spectra of decontamination strippable coatings.

The FT-IR spectra, illustrated in Figure 2, display the variation of the characteristic peaks of the decontamination strippable coatings with the increase in SA concentration.

The O–H stretching vibrations at 3293 cm^−1^ were slightly shifted to 3288 cm^−1^, probably due to the supplementary hydrogen bonds brought by increasing the amount of SA added in the decontamination solutions [35]. By increasing the concentration of SA, the two peaks associated with the asymmetric O–C–O stretching vibrations at 1630 cm^−1^ and 1575 cm^−1^ merged into a singular peak visible at 1610 cm^−1^, confirming that the interaction between SA and the other components of the decontamination films occurs also trough the carboxylic groups. The intensity of the characteristic peak for bentonite (attributed to Si–O stretching vibrations visible at 1029 cm^−1^) decreased with the addition of SA. A clearer indication of the interaction between the Si-OH group from the nanoclay and SA can be highlighted through a more visible peak, at 994 cm^−1^, also given by the Si–O stretching vibrations. The specific peak for mannuronic acid sequence of SA was visible at 820 cm^−1^, being slightly shifted in comparison with pure SA, due to the interactions established between SA and the other components, mainly hydrogen bonds formation. The peak at 850 is due to Si–O–Si vibrations, also slightly shifted in comparison with pure bentonite spectra.

**Figure 3:**
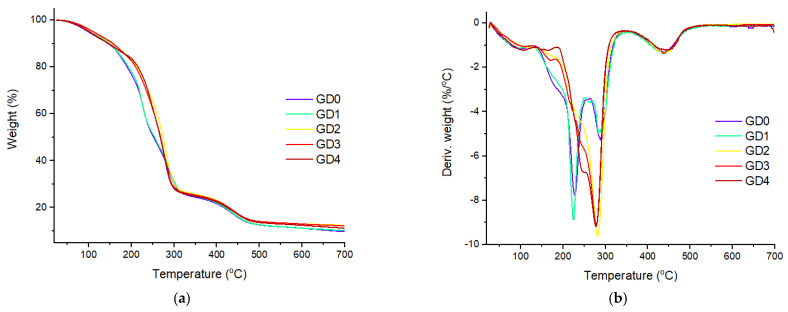
TGA (**a**) and DTG (**b**) plots of decontamination strippable coatings.

The thermal and mechanical properties of the decontamination strippable coatings obtained by employing different concentrations of SA were investigated to establish the optimal composition for these strippable coatings designed for surface decontamination. Figure 3 illustrates the results of the TGA analysis performed on the synthesized materials for the assessment of their thermal stability.

As can it be observed in Figure 3a, all the strippable films exhibited a first weight-loss of approximately 8% (up to approximately 140 °C), probably due to the loss of the residual water in their composition. Additionally, the results confirm the thermal stability of the polymeric nanocomposite films up to 140–150 ℃, after which a second weight-loss stage is registered varying from 15% (GD-0, GD-1, and GD-2) to 7% (GD-3 and GD-4). Therefore, the addition of SA led to a smaller weight-loss for the material in this temperature range. As it can be noticed in Figure 3b, the peak at 220 ℃, assigned to the third weight loss step, is slightly shifted to higher temperatures in the case of GD-2, GD-3, and GD-4 samples; thus, the increase in SA concentration leads to a slightly increased thermal stability for the samples, due to the higher number of hydrogen bonds established between SA and PVA [36]. 

**Figure 4:**
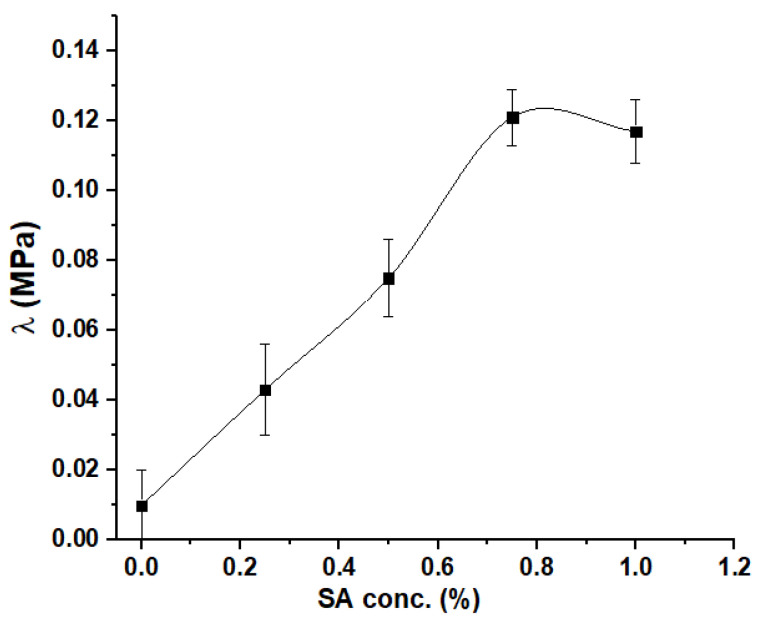
Correlation between the SA concentration and the mechanical properties of the films.

Table 2 summarizes the results obtained from tensile tests. According to these results, the strippable films containing SA possess a higher elasticity. Figure 4 describes the correlation between the SA concentration and the mechanical properties of the nanocomposite strippable films. The value of the elastic modulus (λ = σ/ε) increases with the addition of SA, up to a concentration of approximately 0.8% SA, and then starts to decrease. Considering these results, an optimum amount of SA (approximatively 0.7–0.8%) permits the reduction in PVA concentration, thus affording decontamination strippable coatings with a lower environmental and superior mechanical and thermal properties. 

Thus, based on the above-mentioned analyses, it can be observed that GD-3 possess remarkable thermal properties and adequate mechanical properties for this type of application. Therefore, the solution containing 0.75% Na-Alginate (GD-3) was chosen as the basic polymeric matrix for the decontamination solutions containing also complexing agents. Thus, starting from this point, the following tests were performed only on the blank solution (GD-0), the solution with optimal level of alginate (GD-3), and the solutions comprising both alginate and chelating agents (GD-3-PBTC and GD-3-IDS). 

**Figure 5:**
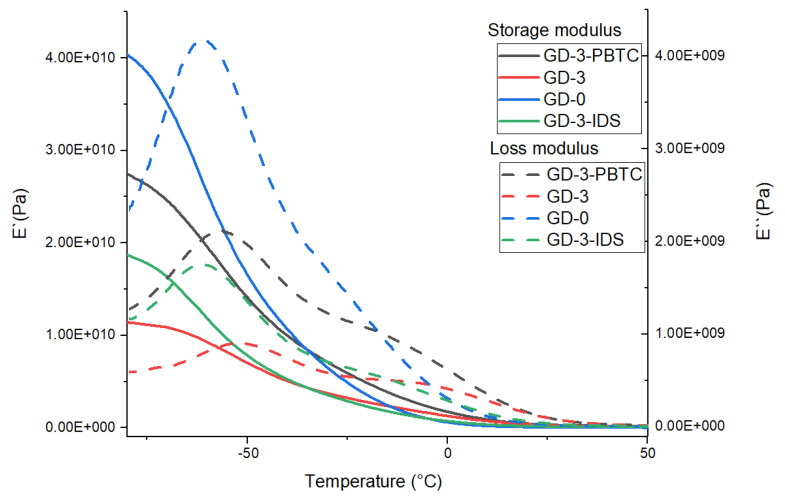
Loss and storage modulus of strippable coatings.

The investigation of viscoelastic behavior is important for a proper design of the strippable coatings. Considering the results obtained from the tensile tests, which concluded that GD-3 displayed the best mechanical characteristics, four types of polymeric composites were selected to be subjected to DMA analysis: GD-0, GD-3, GD-3-PBTC, and GD-3-IDS. Figure 5 illustrates the comparative graph between these four types of decontamination polymeric composite films.

DMA results revealed distinct viscoelastic behavior of the cured strippable coatings depending on the chelating agents employed in the decontamination solutions. An adequate viscoelasticity is important for the peeling process of these coatings because their components should facilitate the final removal of the polymeric film from the decontaminated surface, while ensuring a high decontamination performance. When a stress is applied to a viscoelastic material [37] such as our decontamination coatings, during the peeling process, some parts of the long polymer chain change their position and when the stress is taken away, namely when the coating is completely peeled off, the accumulated back stresses will bring the polymeric nanocomposite film to its original shape, because, theoretically, the polymer chains will return to their initial position. When a stress is applied, the strippable coatings possessing higher viscoelasticity (higher storage and loss modulus values) will undergo a more significant molecular rearrangement. Whereas elasticity (described by the variation of storage modulus) is related to bond stretching, viscosity (described by the variation of loss modulus) is related to the diffusion of molecules inside an amorphous material [38]. From Figure 5, we can conclude that storage and loss modulus decrease in the following order: GD-0 > GD-3-PBTC > GD-3-IDS > GD-3, probably due to the different number of hydrogen bonds established inside the polymeric nanocomposite and depending on different strengths of these hydrogen bonds. The formed hydrogen-bonds network is responsible for the mechanical and structural properties of a material. Therefore, according to the values obtained for storage and loss modulus, it can be noticed that the strippable coatings without alginate and chelating agent exhibited the highest viscoelasticity, the one with alginate displayed the lowest viscoelasticity, while the ones containing IDS and PBTC display improved qualities compared to GD-3 [39].

**Figure 6:**
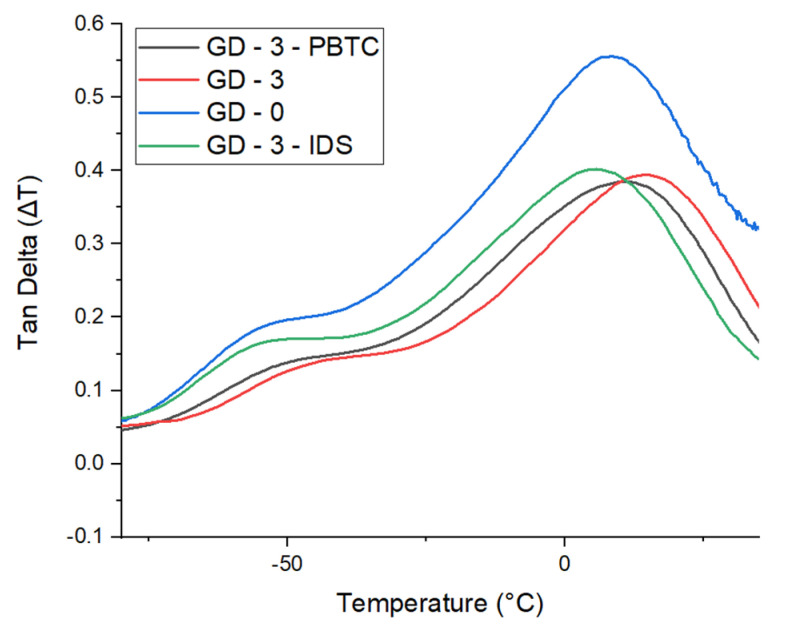
Tan Delta of strippable coatings.

The thermal properties of the polymeric nanocomposite films employed for decontamination were determined using the DMA technique because it can detect molecular relaxations, such as glass transition temperatures (Tg), with a higher accuracy than DSC or DTA [40,41]. According to the tan delta (δ) plots (Figure 6), the strippable coatings displayed an apparent double glass transition temperature (Table 3). This property was predictable because neat PVA (the polymer matrix employed in these decontamination nanocomposite films) shows typical behavior of a semi-crystalline polymer with two transition regions as well: T_g1_ ≈ 30–60 °C and T_g2_ ≈ 80–150 °C [39,42]. Our PVA nanocomposite peelable films possess significantly lower glass transition values due to their higher flexibility given by glycerol and the other additives. Therefore, the lower temperature amorphous glass transition, T_g1_, varies from −61.8 °C to −51.6 °C and could be associated with the glass-rubber transition of the amorphous phase of the polyvinyl alcohol composites while the higher amorphous glass transitions, T_g2_, varies from 5.7 °C to 14.6 °C and could be assigned to the relaxation of the PVA crystalline domains, representing the temperature range where amorphous, rubbery, and crystalline domains coexist [39,42,43]. Alginate and the chelating agents employed in these decontamination coatings also influence glass transition due to the interactions with the polymeric chain. The hydrogen bonds established between the hydroxyl groups of PVA and the other components of the strippable coating, influence the polymer chains mobility; therefore, both glass transition temperatures will be different for each strippable coating, depending on their composition, as can be observed in Table 3.

After characterizing the peelable coatings employed for decontamination, the next objectives consisted of the evaluation of the decontamination efficiency. This goal was accomplished through various analytical techniques: SEM-EDS, AAS, and radiation measurements.

**Figure 7:**
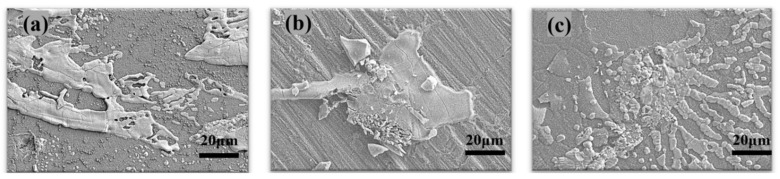
SEM images of cesium surface contamination patterns on different types of surfaces (before decontamination): (**a**) mirror-finish stainless steel, (**b**) grinded-finish stainless steel, and (**c**) etched-finish stainless steel.

The morphological evaluation of the targeted surfaces, prior- and post-decontamination, offered a preview on the decontamination efficiency through the qualitative information offered by this technique. Since decontamination performances also depend on the type of surface that requires decontamination, SEM/EDS analyses were carried out to observe the contaminant behavior on three types of stainless-steel surfaces: mirror-finish (SSMF), grinded-finish (SSGF), and etched-finish (SSEF). SEM analyses are made in a controlled environment, which cannot be contaminated with radioactive materials; thus, for these analyses, we employed ^133^Cs as simulant for ^137^Cs. For the controlled contamination of the above-described surfaces, a 0.005 M Cs_2_SO_4_ aqueous solution was utilized. The dispersion-pattern of the contaminant on each surface depends on the roughness of the employed material. As shown in Figure 7a, when applied to a mirror-finish metallic surface, contaminant droplets tend to clump in one place. The molecules of these aqueous droplets are held together by strong cohesive forces that are intermolecular attractive forces, which leads to the formation of large crystals that exhibit low-adhesion forces towards the mirror-finish metallic surface; therefore, they were easily removed using strippable coatings method. When surface roughness increases, the contaminant droplets tend to spread much more along the axes of the cracks: linear direction on grinded-finish stainless steel (Figure 7b), and dendritic direction on etched-finished stainless steel (Figure 7c), thus penetrating the interstices of the material.

For the controlled contamination process, we tried to reproduce cesium contamination at levels representative of those found in the nuclear industry, namely we started with 5 mM (C1) of Cs_2_SO_4_ aqueous solution, a similar concentration with those mentioned in the literature [7,44]. After being subjected to the controlled contamination process, stainless-steel samples were further analyzed by the EDS technique, the mean values, and standard deviations for the determined and calculated values are reported in Table 4. The results obtained through SEM-EDS technique for the metallic samples subjected to controlled “artificial in-depth contamination”, with C1 at 700 °C, did not render major differences in terms of decontamination factors obtained, since cesium could not be detected on the tested surface after decontamination. The only difference, when compared to superficial contamination with C1, consisted of the aspect of the contaminated surface after being subjected to the thermal treatment and the visible solid residues entrapped in the polymeric films (Figure S2 Supplementary Material). Since this attempt led to quite similar results with the samples subjected to “superficial” contamination, only the results obtained for superficial contamination are worth to be further detailed. 

As expected, the relative atomic concentration (at.%) [45] was higher on mirror-finish surfaces where the droplets tended to agglomerate, and was lower in the cases of grinded-finish and etched-finish surfaces, according to the increasing number of surface imperfections, as, in these cases, the contaminant spread on the higher surface while also entering the pores and cracks of the metallic sheets, leading, at the same time, to in-depth contamination.

**Figure 8:**
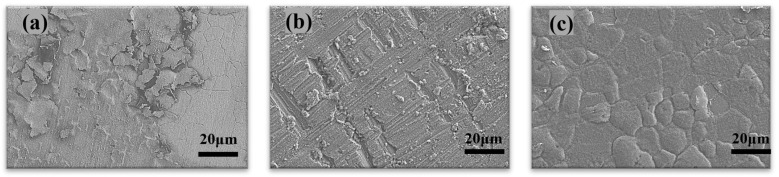
SEM images of surfaces (after decontamination): (**a**) mirror-finish stainless steel, (**b**) grinded-finish stainless steel and, (**c**) etched-finish stainless steel. (These surfaces were contaminated with 0.005 M Cs_2_SO_4_ (C1) solution and decontaminated with GD-3-IDS.)

After the controlled contamination step, the decontamination solutions were poured over the contaminated surfaces and allowed to dry. All four types of decontamination solutions (GD-0, GD-3, GD-3-PBTC, and GD-3-IDS) were tested on mirror-finish, grinded-finish, and etched-finish stainless steel samples. Figure 8 illustrates the stainless-steel surfaces after the peeling process. Even though some polymer traces, probably resulting from the peeling process, are slightly observable, cesium crystals were not visible anymore. Thus, this information can serve as a screening method for the preliminary performance evaluation of the strippable films, thus confirming that all the four strippable coatings can be considered efficient for this type of surfaces, because the amount of residual cesium was undetectable after decontamination. Even if supplementary investigations were performed to prove and quantify the decontamination efficiency, SEM-EDS analysis offered, however, a reasonable preview of decontaminated surfaces.

**Figure 9:**
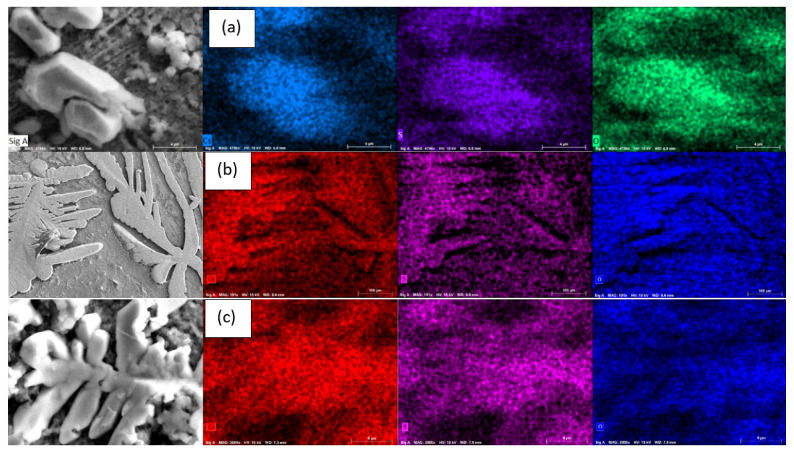
Exemplification of SEM images after decontamination and EDS elemental mapping of Cs, S, and O (the metallic surfaces: (**a**) brass surface (BS), (**b**) galvanized metal surface (GM), and (**c**) copper surface (CS) contaminated with 0.05 M Cs_2_SO_4_ (C2) solution and decontaminated with GD-3-IDS.

SEM-EDS analysis was also performed at higher contamination levels compared to those found in the literature, for a rough estimation of the upper limit of Cs retention in the polymeric coatings. For this purpose, two supplementary concentrated Cs_2_SO_4_ aqueous solutions (0.05 M (**C2**) and 0.5 M (**C3**)) were employed to find a correlation between the amount of Cs on the metallic surface and the upper limit of Cs retention in the polymeric coatings. The behavior of these strippable coatings on other types of metallic surfaces was also evaluated using galvanized metal (GM), brass (BS), and cooper substrates (CS). These three types of metallic surfaces (GM, BS, and CS) were contaminated with 0.005 M (**C1**), 0.05 M (**C2**), and 0.5 M (**C3**) Cs_2_SO_4_ aqueous solutions. Contamination and decontamination were performed according to the same procedure (described for **C1**) for all samples, but different amounts of residual cesium were still detected on some of the metallic surfaces, as can be observed in Table 5. Successful decontamination results were obtained for the metallic coupons contaminated with the lowest concentration of Cs (**C1**). For **C1**, after peeling the strippable decontamination coatings, undetectable amounts of residual cesium or a maximum of 0.66 Cs (at. %) for SC2-PBTC were measured for GMS. When the level of contamination was increased, using 0.05 M (**C2**) and 0.5 M (**C3**) Cs_2_SO_4_, decontamination efficiency seemed to decrease. From Table 5, it can be noticed that after decontamination, residual Cs levels increased, according to the contamination level (C3 > C2 > C1), but the residual levels are also influenced by the type of the surface and the decontamination solution employed. Even so, for the usual contamination levels (**C1**) found in the literature for the nuclear industry, GD-3-IDS displayed the best decontamination performances, for all the tested surfaces; because in this case, the residual cesium was undetectable after decontamination. Figure 9 showed that employing different types of metallic surfaces led to Cs_2_SO_4_ crystals with distinct shapes, depending on the interaction with these metals. Therefore, on brass surfaces, the crystals have an elongated hexagonal shape, and on galvanized metal and copper surfaces, the crystals have a dendritic shape. When comparing the SEM images from Figure 9 with the ones illustrated in Figure 8, corelated with the data from Table 5, it can be noticed that as expected, the polymeric nanocomposite films become less efficient at higher levels of contamination (concentrations higher than 0.005 M). Thus, if this extreme situation were to be encountered in real contamination circumstances, several repeated decontamination procedures would be required for the complete removal of the contaminant. Another solution could be to increase the concentration of the chelating agent, but this might induce modifications in the properties of the strippable nanocomposite film, which is not desirable because it might reduce its performance. 

It is useful to reiterate that the SEM-EDS method was employed only as screening method, the data obtained being useful only as preliminary results and complementary information for further investigations. Supplementary analyses with higher accuracy, which offer the possibility of better quantifying the contamination/decontamination levels, calculated according to AEP-58 NATO standard, were further employed (AAS technique and measurement of the activity of the radioactive materials) and are described in the following section of the study.

### Decontamination Tests

**Figure 10:**
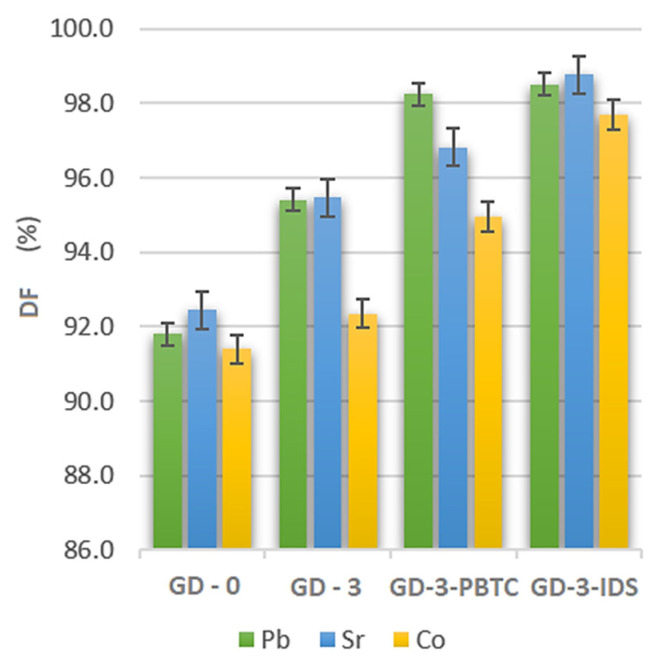
Efficacy of the strippable coatings for toxic metals removal illustrated (DF calculus based on the concentration of metal before and after decontamination).

Heavy metal uptake capacity of the strippable coating was evaluated by applying the decontamination solutions on glass surfaces contaminated with lead (Pb), strontium (Sr), and cobalt (Co), employed in this experiment as simulants for their radioactive analogues, were also subjected to the same procedure of analysis. Atomic adsorption spectrometry (AAS) was utilized to assess the concentration of “toxic metals” before and after decontamination. The data obtained from AAS analysis were utilized to calculate the decontamination efficacy of each solution, for every metal. The decontamination factor, DF = 100 (C_0_ − C_f_)/C_0_ (DF is the decontamination factor, C_0_ is the initial metal concentration, and C_f_ is the final concentration) was calculated. Figure 10 illustrates a comparative plot comprising the results obtained for glass surfaces contaminated with Pb, Sr, and Co. 

For each metal tested, different decontamination degrees were reached, depending on the intensity of the interaction between the targeted metal and the surface on which it was deposited and the interaction with the components of the decontamination solutions. As it can be observed in Figure 10, the reference sample (Bk0), exhibited lower decontamination efficiency for all the three metals tested, because, in this case, metal uptake involved only the physical interactions established between the nanoclay adsorbent and the contaminant. The metallic contaminant was entrapped and fixed in the matrix of the nanocomposite, and it was removed along with the exfoliation of the dry nanocomposite film. The influence of SA on the decontamination efficiency can be deduced from the values obtained and presented in Figure 10 with Alg label attributed to GD-3 decontamination solution. The chelating ability of sodium alginate significantly improved the decontamination performances, obtaining higher DF for all the metals tested. The “green chelates”, IDS and PBTC had a significant positive influence on the decontamination efficacy of the solutions, confirmed by the results presented in Figure 10 (Alg-PBTC refers to GD-3-PBTC and Alg-IDS refers to GD-3-IDS). These results showed that IDS led to higher decontamination factors (as anticipated from the preliminary data obtained from SEM-EDS analysis). Therefore, these experiments proved the influence of each of the components on the performances of the decontamination solutions, showing that GD-3-IDS, especially, can be successfully employed for the removal of heavy metals from the contaminated surfaces.

**Figure 11:**
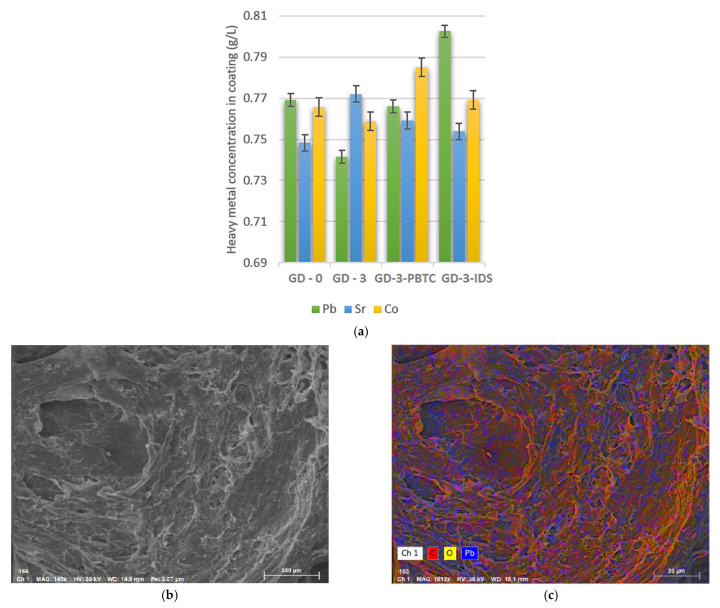
(**a**) Concentration of toxic metals found in the peeled nanocomposite film after decontamination; (**b**) SEM image of the nanocomposite film after decontamination of lead; (**c**) EDS image displaying C, O, and Pb elements present in the film obtained after decontamination of lead.

Figure 11 offers information about the presence and abundance of the metals in the nanocomposite coatings after decontamination. However, because the process of obtaining clear AAS solutions required intermediary steps in this case (described in Methods Section 2.2), we cannot retrieve the expected concentration of metal in the values obtained, probably because a part of the metallic contaminant was fixed by bentonite. Even so, most of the contamination can be retrieved in Figure 11, while the results summarized in Figure 10 showed that on the decontaminated surface the toxic metals were only found as traces. 

**Figure 12:**
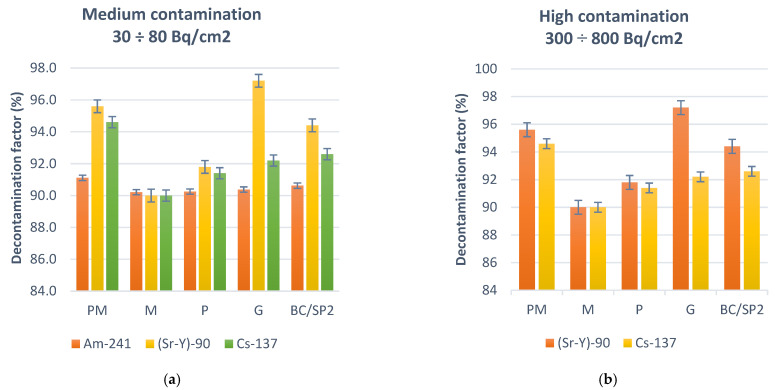
Decontamination factors obtained for GD-3-IDS decontamination solution in two distinct scenarios: (**a**) medium contamination and (**b**) high contamination (PM—painted metal; M—metal; P—plastic; G—glass; and BC/SP2—CBRN protective material).

The most relevant tests in this study are represented by the decontamination investigations performed on radioactive materials. For this purpose, we used five different types of surfaces (metal-M, painted metal-PM, plastic-P, glass-G, and CBRN protective material—BC/SP2) and three radioactive solutions to generate alpha (^241^Am), beta (^90^(Sr-Y)), and gamma (^137^Cs) contamination. The decontamination solution employed for these experiments was GD-3-IDS, due to its remarkable efficiency, sustained by the previous results obtained in this study. The decontamination method herein described is in accordance with AEP-58 NATO standard. This military standard also establishes the minimal requirements for a new decontamination method to be considered efficient: the decontamination factor must have a value higher than 90% [4]. The results depicted in Figure 12 demonstrate that this criterion was successfully reached by GD-3-IDS decontamination solution. As can be seen, all the decontamination factors obtained indicated values above 90%, thus demonstrating that GD-3-IDS polymeric nanocomposite coatings efficiently removed radioactive contamination.

In the first scenario described in Figure 12 implied a medium contamination level of 30 ÷ 80 Bq/cm^2^ while the second scenario implied a high contamination level of 300 ÷ 800 Bq/cm^2^. ^241^Am was tested only in medium contamination scenario, due to safety reasons. From Figure 12, it can be noted that decontamination efficiency of GD-3-IDS varies with the type of targeted surface, probably due to the different adherence of the polymeric matrix to each substrate and with the type of radionuclide employed for contamination. This can be explained by the different interactions established between the contaminant and the contaminated surface, and between the contaminant and the components of the decontamination solution (GD-3-IDS).

The advanced performances of the decontamination solutions developed in this study are sustained especially by the high contamination scenario, because even at this high contamination level, GD-3-IDS still managed to reach the 90%DF imposed by NATO standards. Therefore, even if the values obtained for DF were lower in high contamination than in the case of medium contamination, the strippable nanocomposite films can still be considered efficient in removing ^241^Am, ^90^(Sr-Y), and ^137^Cs contaminants. 

## 4. Conclusions

Novel environmentally friendly surface decontamination solutions were successfully prepared based on PVA/SA/GLY/BT/IDS or PBTC aqueous mixtures. After deposition and during drying the solutions were capable to entrap heavy metals and radionuclide contaminants using physical and chemical processes by generating a composite polymeric film with good wetting capability on a large variety of solid surfaces displaying peeling-off capacity.

Chemical, mechanical, and thermal characterization of the polymeric films were performed using FTIR, tensile tests, TGA, and DMA techniques showing the influence of each component and allowing further optimization and selection of the formula for decontamination performance tests.

Controlled contaminations were performed on various surfaces metals (with different finishes), painted metal, plastic, glass, and CBRN protective material, according to NATO standard AEP-58 and using typical concentrations (or above) for contaminated sites found in the literature. 

The decontamination effectiveness was first evaluated in a qualitative manner using SEM-EDS techniques followed by a quantitative approach employing AAS and surface activity measurements on live radioactive agents. The influence of SA, IDS, PBTC concentrations, and surface type over the DF was also emphasized. The presence of sodium alginate, and especially of chelating agents IDS/PBTC, decisively improves the decontamination factor. 

Solution GD-3-IDS containing 5% PVA, 1% BT nano-clay, 2.5% glycerol, 0.75% sodium alginate, and 1% IDS chelating agent showed the best results as having decontamination factors that overpassed the DF imposed by NATO standards [4], DF being ≥90% for all surfaces tested and for the highest contaminations.

## Figures and Tables

**Table 1:** Composition of the gel-forming decontamination solutions.

Sample ID	SA (Weight %) *	CA (Weight %) *
GD-0	0	0
GD-1	0.25	0
GD-2	0.5	0
GD-3	0.75	0
GD-4	1	0
GD-3-PBTC	0.75	1
GD-3-IDS	0.75	1

* % calculated from the total mass of the decontamination solution.

**Table 2:** Mechanical properties nanocomposite films.

Sample ID	Measured Values		Calculated Values	
	L_b_ (mm)	F_m_ (N)	σ = TS_b_ (MPa)	ε = E_b_ (%)
GD-0	182.1 ± 26.6	23.2 ± 3.5	3.6 ± 0.5	363.8 ± 53.1
GD-1	135.6 ± 20.3	28.5 ± 2.7	11.5 ± 1.3	271.2 ± 40.6
GD-2	55.9 ± 25.4	22.3 ± 1.9	8.4 ± 1.1	111.8 ± 50.8
GD-3	39.0 ± 6.5	33.6 ± 0.7	9.9 ± 1.0	82.1 ± 12.9
GD-4	29.5 ± 5.5	18.7 ± 5.9	6.9 ± 1.6	58.9 ± 5.5

F_m_ is the maximum force recorded. Tensile stress, **σ**, was calculated, according to ISO37:2011(E), taking into account **W** (the average width of the narrow part of the specimen, mm) and **t** (the average thickness, mm): σ = F_m_/(W∙t). Tensile strain, **ε**, was calculated, according to ISO37:2011(E), taking into account **L_b_** (length at breaking, mm) and **L_0_** (the initial length, mm): ε = 100∙(L_b_ − L_0_)/L_0_. TS_b_ is the tensile strength at break (tensile stress recorded at the moment of rupture). E_b_ stands for elongation at break. The average values for each sample were reported.

**Table 3:** Characteristic temperatures * for the polymeric films.

Name	T_g1_ (°C)	T_g2_ (°C)
**GD-3**	−51.6	14.6
**GD-3-IDS**	−61.8	5.7
**GD-3-PBTC**	−56.7	10.3
**GD-0**	−61.5	8.7

* Tg_1_ and Tg_2_ correspond to the maximum value of each of the two tan delta peaks.

**Table 4:** EDS analysis of the control sample.

No.	Type of Surface	Cs (at. %) *	Cs (wt.%) *	Cs Abs. Error (%) **
1	SSMF	15.23	58.48	2.42
2	SSGF	10.12	40.22	0.83
3	SSEF	3.74	15.74	0.3

* Detailed in Supplementary Material in Figures S3–S5 and Tables S2–S4, respectively. The values reported for wt% and at% were calculated using the Bruker ESPRIT QUANTAX software of the scanning electron microscope. ** Abs. Errors (%) were calculate by Bruker ESPRIT QUANTAX software at 3 sigma for a single determination.

**Table 5:** EDS—comparative evaluation of the remnant contaminant after decontamination (means not detectable).

GD-3-IDS									
Concentration	C1			C2			C3		
Surface	GM	BS	CS	GM	BS	CS	GM	BS	CS
Cs (at. %) experimental	-	-	-	2.97 ± 0.36	1.29 ± 0.21	3.01 ± 0.15	9.47 ± 0.46	4.96 ± 0.36	5.22 ± 0.35
Cs (wt.%) calculated	-	-	-	10.83	8.37	6.09	37.23	28.98	30.06
GD-3-PBTC									
Concentration	C1			C2			C3		
Surface	GM	BS	CS	GM	BS	CS	GM	BS	CS
Cs (at. %) experimental	0.66 ± 0.12	-	-	2.46 ± 0.28	2.67 ± 0.19	3.95 ± 0.18	4.79 ± 0.37	6.45 ± 0.34	9.86 ± 0.42
Cs (wt.%) calculated	2	-	-	8.97	9.29	9.09	18.25	19.68	40.6

## Data Availability

The data presented in this study are available on request from the corresponding author.

## Supplementary Materials

The following are available online at https://www.mdpi.com/article/10.3390/polym13234194/s1. Figure S1—Different types of “polishing” for the stainless-steel coupons employed for decontamination tests prior to contamination step; Figure S2—Contamination (a) immersion in Cs_2_SO_4_ aqueous solution (C1 = 0.005 M); (b) “in-depth” contamination at 700 °C; (c) metallic coupons after in-depth decontamination; and Decontamination processes (d) applying one of the decontamination solutions; (e) peeled film; (f) different types of metallic samples prepared for SEM-EDS analysis after decontamination; Table S1—AAS instrumental parameters; Figure S3—EDS spectrum for SSMF contaminated with Cs; Table S2—Values obtained from EDS analysis for SSMF contaminated with Cs; Figure S4—EDS spectrum for SSGF contaminated with Cs; Table S3—Values obtained from EDS analysis for SSGF contaminated with Cs; Figure S5—EDS spectrum for SSEF contaminated with Cs; Table S4—Values obtained from EDS analysis for SSEF contaminated with Cs.
