# Supplementary material for: Strippable Polymeric Nanocomposites Comprising “Green” Chelates, for the Removal of Heavy Metals and Radionuclides
Source: Polymers (Basel). 2021 Nov 30;13(23):4194. doi: 10.3390/polym13234194 (PMC8659526; doi:10.3390/polym13234194)
Supplement: Supplementary file 1 [file polymers-13-04194-s001.zip › polymers-1451526-supplementary.pdf]

Supplementary Materials

For article

Strippable polymer nanocomposite comprising "green" chelates, for the removal of heavy metals and radionuclides

Gabriela Toader [1,‡], Daniela Pulpea [1,‡], Traian Rotariu [1,*], Aurel Diacon [2], Edina Rusen [2,*], Andreea Moldovan [1], Alice Podaru [1], Raluca Ginghină [3], Florentina Alexe [3], Ovidiu Iorga [3], Sorina Aurora Bajenaru[3], Mihai Ungureanu[1], Florin Dîrloman[1], Bogdan Pulpea[1] and Lucia Leonat[4]

[1]Military Technical Academy "Ferdinand I", 39-49 George Coșbuc Boulevard, Bucharest, 050141, Romania
[2]Faculty of Applied Chemistry and Materials Science, University 'Politehnica' of Bucharest, 1-7 Gh. Polizu Street, Bucharest, 011061, Romania
[3]Research and Innovation Center for CBRN Defense and Ecology, 225 Soseaua Oltenitei, Bucharest, 041327, Romania
[4]National Institute of Materials Physics, 077125, Măgurele, Romania
‡ Contributed equally to this study (co-first authors)
*Corresponding authors: traian.rotariu@gmail.com and edina.rusen@upb.ro

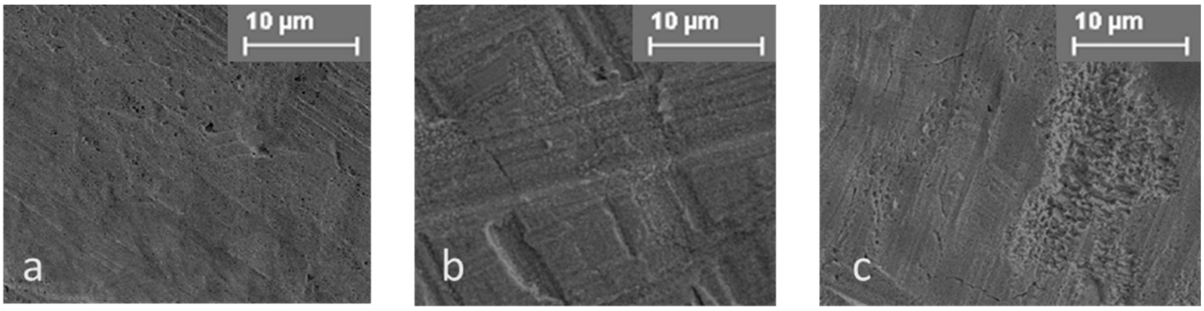

Figure S.1 – Different types of "polishing" for the stainless-steel coupons employed for decontamination tests prior to contamination step: (a) mirror – finish (SSMF); (b) grinded-finish (SSGF); (c) etched-finish (SSEF)

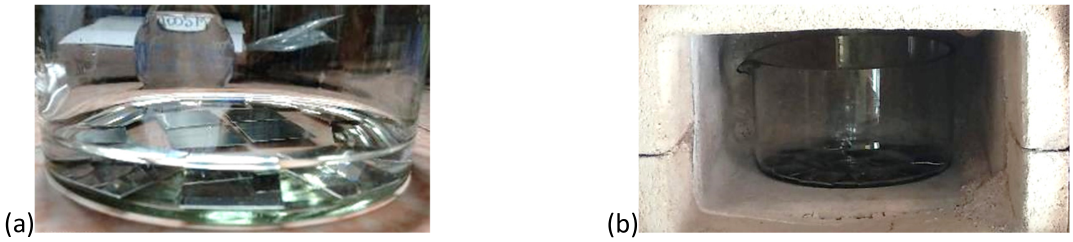

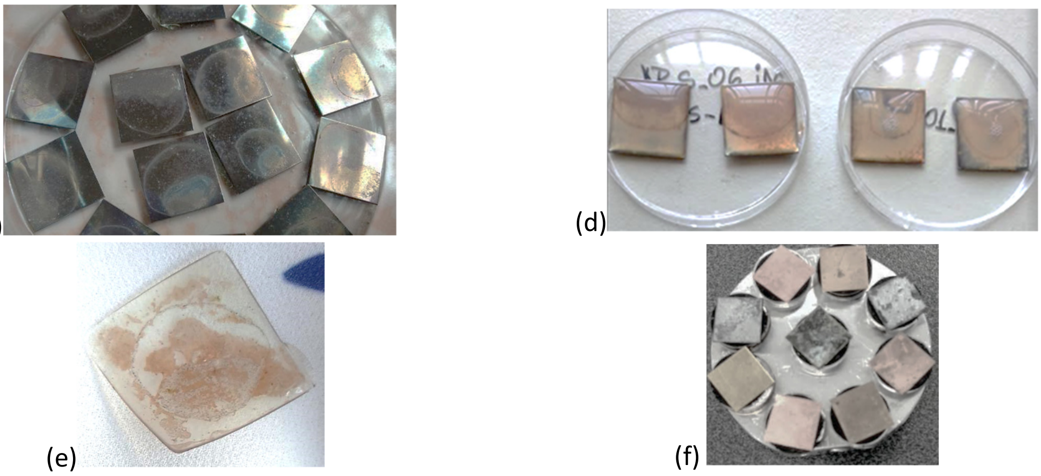

Figure S.2 – Contamination (a) immersion in Cs_2SO_4 aqueous solution (C1 = 0.005M); (b) "in-depth" contamination at 700°C; (c) metallic coupons after in-depth decontamination; and Decontamination processes (d) applying one of the decontamination solutions; (e) peeled film; (f) different types of metallic samples prepared for SEM-EDS analysis after decontamination.

Table S.1 - AAS instrumental parameters

**Element**	**Pb**	**Co**	**Sr**
Wavelenght(nm)	283.3	242.5	460.7
Lamp	Hollow catode		
Slit Width	0.7L	0.2L	0.7L
READ TIME	5 sec		
Graphite tube	Pyrolytic graphite		
**Calibration**			
**Standards**	Conc. (μg/L)	Conc. (μg/L)	Conc. (μg/L)
Std.1	5	5	3
Std.2	10	10	6
Std.3	15	15	9
Std.4	20	20	12
Std.5	25	25	15
Calibration Method	zero intercept. linear		
Techique	AA-BG	AA-BG	AA
Signal processing	Peak Area		
Sample volume	20 μL		
Replicate	3		
Modifier /volume	Pd/Mg(NO3)2/ 5 μL	Mg(NO3)2 / 5 μL	Pd/Mg(NO3)2 5 μL

Furnace program for Pb, Co, Sr

Step	Temp (°C)	Ramp time (s)	Hold time (s)	Internal flow (ml/min)	Gas type
1	110	1	30	250	Argon
2	130	15	30	250	Argon
3	850	10	20	250	Argon
4	1800	0	5	0	Argon
5	2450	1	3	250	Argon

Injection temperature - 20 (°C). Read step - 4

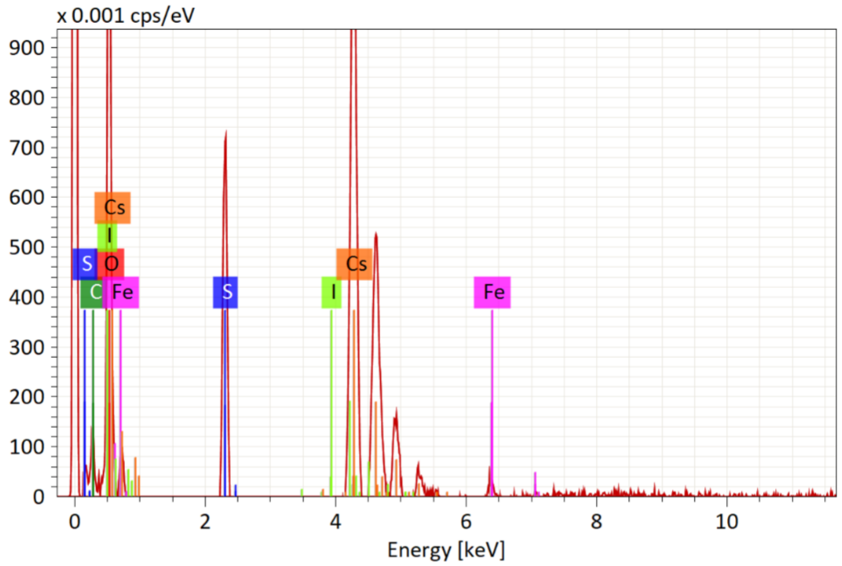

Figure S.3 – EDS spectrum for SSMF contaminated with Cs

Table S.2 – Values obtained from EDS analysis for SSMF contaminated with Cs

Element	At. No.	Netto	Mass [%]	Mass Norm. [%]	Atom [%]	abs. error [%] (3 sigma)	rel. error [%] (3 sigma)
C	6	717	2.14	4.60	13.27	1.20	55.92
O	8	8652	13.88	29.87	64.63	5.14	36.99
S	16	6116	2.53	5.45	5.88	0.34	13.53
Fe	26	318	0.74	1.59	0.99	0.14	18.87
Cs	55	23149	27.18	58.48	15.23	2.42	8.90
		**Sum**	**46.47**	**100.00**	**100.00**		

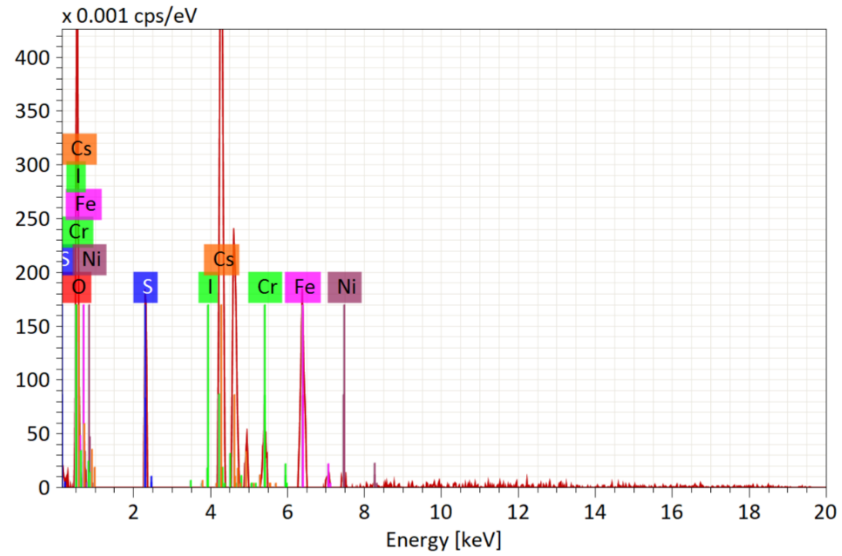

Figure S.4 – EDS spectrum for SSGF contaminated with Cs

Table S.3 – Values obtained from EDS analysis for SSGF contaminated with Cs

Element	At. No.	Netto	Mass [%]	Mass Norm. [%]	Atom [%]	abs. error [%] (3 sigma)	rel. error [%] (3 sigma)
O	8	2867	8.05	35.45	74.05	3.39	42.14
S	16	1281	0.59	2.62	2.73	0.14	23.33
Cr	24	681	0.71	3.15	2.02	0.13	18.53
Fe	26	2223	4.03	17.72	10.60	0.39	9.79
Ni	28	58	0.19	0.85	0.48	0.09	46.98
Cs	55	9430	9.14	40.22	10.12	0.83	9.09
		**Sum**	**22.72**	**100.00**	**100.00**		

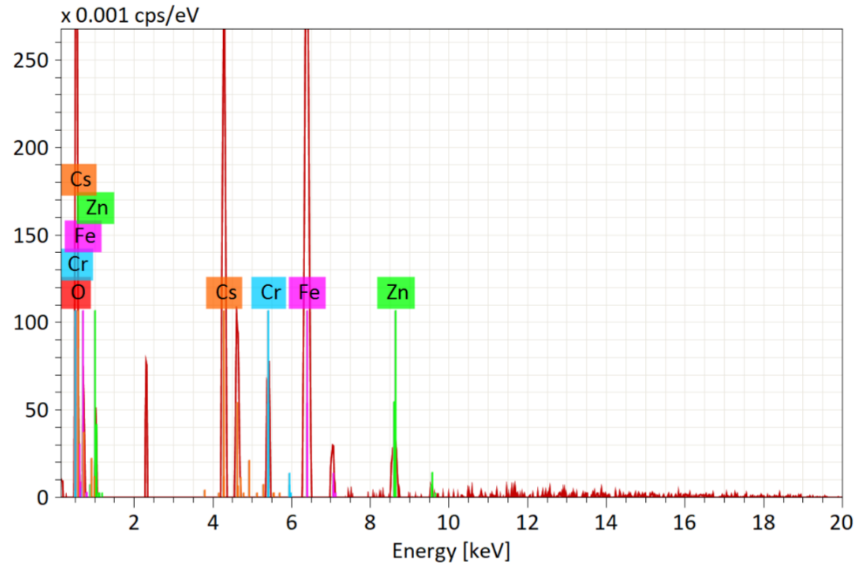

Figure S.5 – EDS spectrum for SSEF contaminated with Cs

Table S.4 – Values obtained from EDS analysis for SSEF contaminated with Cs

Element	At. No.	Netto	Mass [%]	Mass Norm. [%]	Atom [%]	abs. error [%] (3 sigma)	rel. error [%] (3 sigma)
O	8	2968	6.09	35.24	69.54	2.57	42.24
Cr	24	737	0.48	2.79	1.70	0.11	23.57
Fe	26	4764	5.64	32.65	18.46	0.52	9.26
Zn	30	490	2.35	13.58	6.56	0.26	11.27
Cs	55	4069	2.72	15.74	3.74	0.30	11.02
		**Sum**	**17.28**	**100.00**	**100.00**		
